# Mg–Al LDH/Tryptophan Nanoparticle System Improves Depressive-like Behavior in Rats

**DOI:** 10.1021/acsomega.5c06491

**Published:** 2025-09-15

**Authors:** Juliana P. S. Nascimento, Taiana C. V. S. Carvalheiro-Simas, Arnold Ferreira Janssen, Maria Luane de S. da Silva, Juliana C. Valente, Mario L. Barreto de Jesus, Flávia G. Silva, Enéas A. Fontes-Júnior, Waldeci Paraguassu, Cláudio M. R. Remédios, Kelson do Carmo Freitas Faial, Paulo R. M. Sousa, José Rogério A. Silva, Jerônimo Lameira, Cristiane S. F. Maia, Carla Carolina F. Meneses, Cláudio N. Alves

**Affiliations:** † Laboratory of Natural Resources and Sustainability of the Amazon, Institute of Exact and Natural Sciences, Federal University of Pará, Belém 66075-110, Pará, Brazil; ‡ Graduate Program in Medicinal Chemistry and Molecular Modeling, Institute of Health Sciences, Federal University of Pará, Belém 66075-110, Pará, Brazil; § Laboratory of Pharmacology of Inflammation and Behavior, Institute of Health Sciences, Federal University of Pará, Belém 66075-110, Pará, Brazil; ∥ Institute of Public Health, Federal University of Western Pará, Santarém 68035-110, Pará, Brazil; ⊥ Laboratory of Nanoscience and Nanotechnology of the Amazon, Faculty of Physics, Federal University of Pará, Belém 66075-110, Pará, Brazil; # Evandro Chagas Institute, 89124 Ministry of Health, Ananindeua 67030-000, Pará, Brazil; ∇ Graduate Program in Sciences and Environment, Institute of Exact and Natural Sciences, Federal University of Pará, Belém 66075-110, Pará, Brazil; ○ Laboratory of Computer Modeling of Molecular Biosystems (CompMBio), Federal University of Pará, Belém 66075-110, Pará, Brazil; ◆ Catalysis and Peptide Research Unit, University of KwaZulu-Natal, Durban 4000, South Africa

## Abstract

The COVID-19 pandemic has intensified global mental health issues, notably anxiety and depression. l-Tryptophan (TRP), a serotonin precursor, has shown promise as a nutritional intervention; however, its clinical application is limited by its poor bioavailability and low blood–brain barrier permeability. In this study, we developed a novel nanohybrid by intercalating TRP into magnesium–aluminum-layered double hydroxides (TRP-LDH) via coprecipitation. The basal reflection shifts and an interlayer space of 1.11 nm confirm the successful intercalation of tryptophan into the LDH structure. FTIR analysis revealed characteristic TRP bands, notably the N–H bending vibration at 1612 cm^–1^, supporting its incorporation into the LDH matrix. In vitro release in phosphate buffer (pH 7.4) demonstrated sustained TRP release for 48 h. From a mechanistic perspective, the LDH matrix likely enhances TRP bioavailability and brain delivery by protecting the amino acid from degradation and facilitating its diffusion across the blood–brain barrier, thus improving serotonergic modulation. In vivo behavioral tests in male Wistar rats (*n* = 8/group) revealed that TRP-LDH (94 mg/kg) significantly increased the struggling time and frequency and immobile frequency and time compared to control and TRP per se subjects in the forced swimming test, a standard model for antidepressant screening drugs, which suggests an antidepressant effect. These findings suggest TRP-LDH as a promising nanohybrid for mood disorder management.

## Graphical abstract

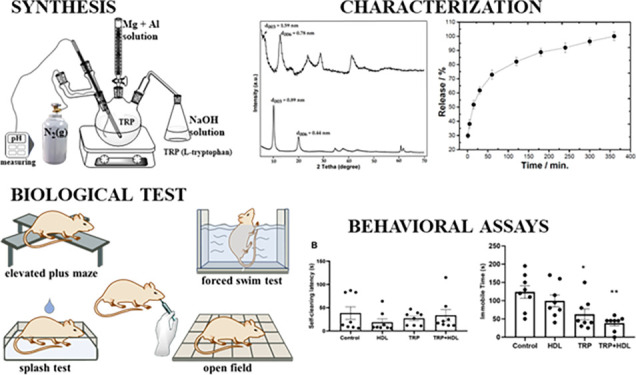

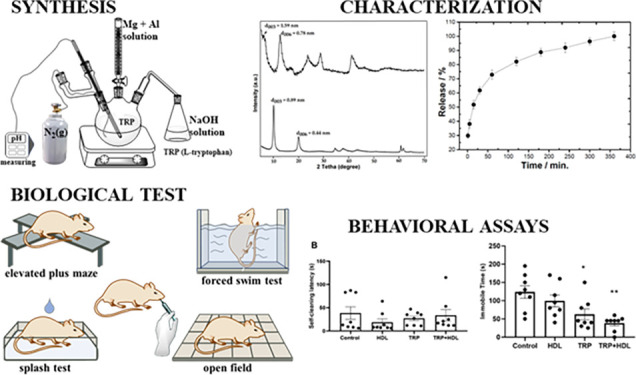

## 1 Introduction

The world is currently experiencing a new wave of health concerns stemming from the long-term effects of the COVID-19 pandemic, most notably a significant increase in mental health disorders such as anxiety and depression. According to the World Health Organization (WHO), approximately 301 million individuals are affected by anxiety disorders, including 58 million children and adolescents, while more than 280 million suffer from depression, with 23 million being young people.^1^ These numbers, which reflect a 25% global increase in the prevalence of anxiety and depression during the first year of the pandemic, underscore the urgent need to expand mental health care services and explore effective, evidence-based therapeutic strategies.^1–4^

Among the emerging therapeutic approaches, nutritional interventions involving l-tryptophan (TRP) have gained attention. TRP is an essential aromatic amino acid obtained exclusively through the diet and is a precursor in the biosynthesis of serotonin (5-hydroxytryptamine, 5-HT), a key neurotransmitter in mood regulation, emotional behavior, and sleep.^5^ Despite its relevance, only 1–5% of absorbed TRP is converted into serotonin in the brain, as the majority is metabolized along alternative pathways.^6^ Nonetheless, studies have shown that TRP supplementation can reduce the symptoms of depression and anxiety. Lindseth et al. demonstrated that participants receiving higher levels of TRP experienced significant reductions in depression, irritability, and anxiety compared to those receiving lower doses.^7^

To overcome limitations related to TRP bioavailability and blood–brain barrier permeability, strategies such as the development of Layered Double Hydroxides (LDHs)/TRP hybrid complexes have been proposed.^8^ LDH nanocarriers improve the solubility and stability of therapeutic molecules, provide protection against enzymatic degradation, and support controlled release, thereby enhancing bioavailability and brain penetration. Previous studies, such as Ferreira Meneses et al.,^9^ demonstrated that LDH intercalation significantly improved the efficacy and biocompatibility of CNS-targeting drugs like indomethacin. These properties make LDHs an attractive platform for delivering TRP, which can more effectively modulate serotonergic pathways. Given that dysregulation acts as a carrier in drug delivery systems due to their structural flexibility, high surface area, and sustained-release capabilities.^10^

LDHs can be synthesized through various methods, such as coprecipitation, ion exchange, and sol–gel techniques, and possess interlayer spaces that facilitate the incorporation of therapeutic agents.^11,12^ When modified into electrically neutral structures, these materials enhance drug loading and release profiles, offering promising solutions for serotonergic systems, which has been implicated in the pathogenesis of numerous psychiatric and neurological disorders, including depression and anxiety,^13,14^ enhancing serotonin levels through targeted supplementation may serve as a valuable adjuvant treatment.

Moreover, LDHs, owing to their biocompatible and versatile structure, have found applications ranging from films to catalysts for the synthesis of valuable compounds.^15^ Their tunable composition and interlayer space also allow the accommodation of fluorescent anions^16^ and various bioactive molecules.^17^ In this context, nanotechnology has emerged as a complementary strategy to improve the stability and delivery of bioactive compounds, and LDHs,^18^ as inorganic nanomaterials, have gained attention for enabling the controlled release of sensitive molecules such as TRP, while providing enhanced protection against degradation in physiological environments.^19^

Thus, this study aims to evaluate the antidepressant-like effects of a novel LDH-TRP hybrid, combining the therapeutic potential of TRP with the controlled release and brain-targeting capabilities of nanostructured carriers. By enhancing stability and modulating the release profile of TRP in physiological environments, LDHs may overcome the limitations of TRP’s poor bioavailability and limited blood–brain barrier permeability. Moreover, the hybrid system could optimize serotonergic modulation at lower doses, potentially reducing side effects and increasing patient adherence. To this end, we synthesized and characterized the TRP-LDH complex and assessed its anxiolytic and antidepressant efficacy through a series of validated behavioral paradigms in a rodent model. This study contributes to the growing field of nanomedicine-based nutritional interventions and offers new perspectives for the development of alternative, noninvasive treatments for mood disorders such as anxiety and depression.

## 2 Materials and Methods

### 2.1 Materials

L-tryptophan (C_11_H_12_N_2_O_2_, 98%), magnesium nitrate hexahydrate (Mg­(NO_3_)_2_·6H_2_O, 98%), aluminum nitrate nonahydrate (Al­(NO_3_)_3_·9H_2_O, 98%), and sodium hydroxide (NaOH, 98%) were purchased from Sigma-Aldrich. Ethanol (CH_3_CH_2_OH, 94–96%) was obtained from Alfa Aesar. All reagents were of analytical grade. Ultrapure water was generated by using an ELGA LabWater PURELAB Option system.

### 2.2 Synthesis of Mg–Al LDH and TRP-LDH Hybrid

Pure Mg–Al LDH and L-tryptophan-intercalated LDH (TRP-LDH) were synthesized via a conventional coprecipitation method, following previously described procedures.^9,20^ In the preparation of TRP/LDH, a mixed aqueous solution of Mg­(NO_3_)_2_·6H_2_O and Al­(NO_3_)_3_·9H_2_O was slowly added to a 1:2 (v/v) acetone–water solution containing l-tryptophan (0.077 mol·L^–1^). The pH was maintained at 10 by the dropwise addition of 1 M NH_4_OH. The resulting gel was aged under vigorous stirring at 60 °C for 24 h in a nitrogen atmosphere to prevent carbonate contamination. The product was collected by centrifugation (1000 rpm, 5 min), washed with a 1:1 ethanol–water solution, and dried in a vacuum desiccator for 72 h. The same procedure was applied for the synthesis of pristine LDH, excluding the amino acid.

### 2.3 Characterization of Mg–Al LDH and TRP/LDH Hybrid

X-ray diffraction (XRD) patterns were collected on a Bruker D8 Advance diffractometer using Cu Kα_1_ radiation over a 2θ range of 20–80°, with a 0.04° step size and 2.8 s acquisition per step. Spectral fitting was conducted using the Peakoc software^21^ with the split pseudo-Voigt function. Fourier-transform infrared (FTIR) spectra were recorded on a Bruker Vertex 70v spectrometer in the 400–4000 cm^–1^ range at 25 °C. Thermal behavior was assessed using thermogravimetric and differential scanning calorimetry (TGA–DSC) on a Netzsch STA 449F3 Jupiter system under nitrogen flow (50 mL·min^–1^), from 25 to 1000 °C at a heating rate of 10 °C·min^–1^. Surface morphology and elemental composition were evaluated by using field-emission scanning electron microscopy (FE-SEM) coupled with energy-dispersive spectroscopy (EDS) (VEGA3, Tescan).

### 2.4 Cumulative In Vitro Release Profile of TRP from TRP-LDH

#### 2.4.1 Release Mechanism Kinetics

To understand the drug release mechanisms of TRP in the TRP-LDH sample, the release profiles were fitted to seven kinetic models. Zero-order release kinetics (eq 1),^22^ First order release kinetics (eq 2),^23^ Korsmeyer–Peppas (eq 3),^23^ Hixon–Crowell (eq 4),^22^ Higuchi (eq 5),^24^ Pseudo-First-Order (eq 6),^25^ and Pseudo-Second-Order (eq 7).^26^

1 $$Q=Q_{0}+K_{0}\cdot t$$

2 $$\ln\left(Q_{t}\right)=\ln\left(Q_{0}\right)+K_{1}$$

3 $$\ln\left(Q_{t}\right)=\ln\left(k_{\mathrm{KP}}\right)+n\mathrm{nl}\left(t\right)$$

4 $${Q_{0}}^{1/3}-{Q_{t}}^{1/3}=K_{HC}\cdot t$$

5 $$Q=K_{H}\cdot t^{1/2}$$

6 $$\ln\left(q_{e}-q\left(t\right)\right)=\ln q_{e}-K_{1}\cdot t$$

7 $$\frac{t}{q_{t}}=\frac{1}{K_{2}q_{e}}+\frac{t}{q_{e}}$$

where *q*_e_ is the equilibrium release amount, *q_t_* is the release amount at any time (*t*), *k* is the rate constant, and *c* is an arbitrary constant.

## 3 Biological Assays

### 3.1 Animals

Male Wistar rats (*n* = 40; 60 days old) were obtained from the Animal Facility of the Evandro Chagas Institute and maintained on a 12:12 h light/dark cycle (lights on 7:00 AM; five animals/cage), with food and water ad libitum, until the beginning of the protocol administration. All procedures were approved by the Ethics Committee on Experimental Animals of the UFPA (license number 5579291117) and followed NIH guidelines for the Care and Use of Laboratory Animals. After the behavioral tests, the animals were anesthetized and sacrificed by cervical dislocation.

### 3.2 Tryptophan Treatment Protocol

Animals were randomly divided into 4 groups (*n* = 8 animals/group). Animals received orally one administration of intercalated tryptophan solubilized in corn oil (94 mg/kg, equivalent to 50 mg/kg of the pure tryptophan), tryptophan per se (50 mg/kg), or corn oil (1 mL/kg; control group).^27^ Negative control received LDH (1 mL/kg). Considering the intercalated tryptophan with the LDH plasma peak, 4 h following the protocol administration, the animals were subjected to the behavioral assays (Figure 1).

**Figure 1:**
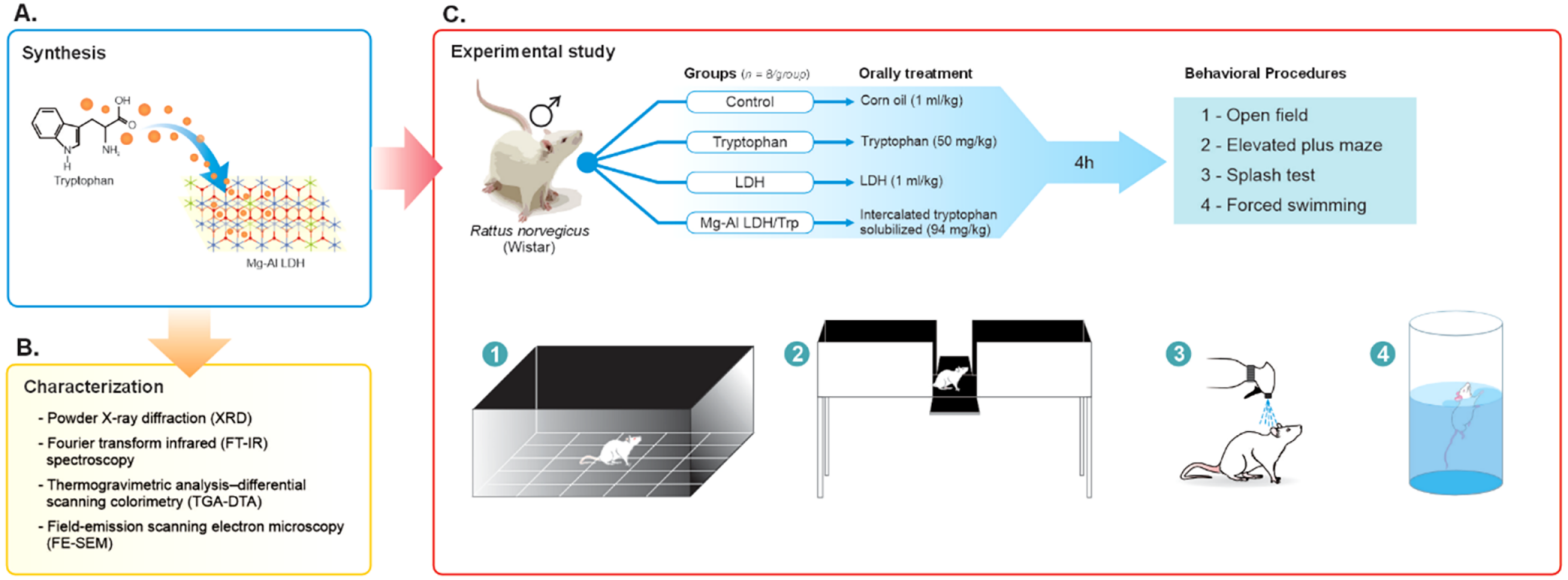
Experimental design.

### 3.3 Open Field Test

The open field test is an important tool for analyzing spontaneous locomotion in rodents, which can be affected by motor function and anxiety-like behavior.^28^ Briefly, animals were placed individually at the center of a black acrylic arena (100 × 100 × 40 cm^3^) and free movement was allowed for 5 min. To evaluate anxiety-like phenotype, the time spent and the distance traveled in the center and periphery of the apparatus were measured.

### 3.4 Elevated Plus Maze

The elevated plus maze consists of a wooden plus apparatus, elevated 50 cm from the floor, with opposite two closed arms (50 × 10 × 40 cm^3^) and two open arms (50 × 10 × 1 cm^3^), surrounded by 1 cm acrylic protection to prevent animals from falling, validated as a gold standard for anxiety-like assessment.^29^ Animals were placed individually at the center of the apparatus, facing one of the closed arms. Animals were allowed to explore the maze for 5 min, and the session was videotaped. The number of entries and time spent in the open arms were automatically measured using the ANY-maze video tracking system (version 4.99, Stoelting Co.). The percentage of open arms entries (%OAE) and time (%OAT) was calculated according to the formula %OAE/OAT = [OAE or OAT/ (OAE or OAT + closed arms entries or time)] × 100.^30^ Anxiety index was calculated according to the formula: anxiety index (%) = 1 – [(open arms time/total time on the maze) + (number the entries to the open arms/total exploration on the maze)/2]. The reduction of the %OAE and %OAT, as well as the increase of the anxiety index percentage, indicates anxiety-like phenotype.

### 3.5 Splash Test

The splash test consists of a behavioral task based on the rat’s preference for sucrose and linked to anhedonia-like behavior.^29^ Animals were placed in individual cages (9 × 7 × 11 cm^3^), and a 10% sucrose solution was sprayed on the dorsal coat, which elicits grooming behavior. The latency to start and spend in the self-cleaning behavior for 5 min was manually recorded as an index of self-care and motivational behavior.

### 3.6 Forced Swimming Test

The forced swimming test consists of a test primarily based on behavioral despair, sensitive to identify motivational behavior (symptom) related to some aspects analogs of depression in humans.^31,32^ The apparatus consists of an acrylic cylinder (30 cm diameter × 50 cm height), filled with water (40 cm high) at a temperature of 23 ± 1 °C. Animals were individually placed in the center of the tank, and free movement was allowed for 5 min. The first 2 min were adopted as a habituation stage. Frequency and time of struggling, swimming, and immobility behaviors were recorded in the last 3 min of the session.^32^ The reduction of struggling and swimming, as well as the increase in immobility time, suggests the depressant-like behavior.

### 3.7 Statistical Analysis

Normality was assessed using the Shapiro–Wilk test. Parametric data were analyzed by on-way analysis of variance (ANOVA) followed by Sidak's multiple comparison test, whereas nonparametric data were analyzed using the Kruskal–Wallis test. The Kruskal–Wallis test followed by Dunn’s multiple comparisons test was used for non-normality values. Results were shown as the mean ± standard error mean (s.e.m.), with *p* < 0.05 adopted as a significant difference between the groups. All statistical analyses were performed by GraphPad Prism 8.0.2 software (San Diego, CA).

## 4 Results and Discussion

### 4.1 Synthesis and Characterization of Particles

The X-ray diffraction (XRD) patterns of the Mg–Al layered double hydroxide (LDH) (Mg–Al–NO_3_^–^) before and after intercalation are shown in Figure 2a. The diffractograms exhibit a series of Bragg reflection peaks characteristic of a single-phase, well-crystallized hydrotalcite-like structure.^9,33,34^ The symmetric and asymmetric reflections at 2θ = 10.02; 19.97 and 60.63° were observed for Mg–Al LDH at (*d*_003_); (*d*_006_) and (*d*_110_) planes (Figure 2a).^35,36^ The full width at half-maximum (FWHM) of the (*d*_003_) reflection was approximately 0.54°, yielding a calculated crystallite size of 16.35 nm along the *c*-axis using the Scherrer equation,^5^ which corresponds to a stacking of approximately 18–19 monolayers.^37^

**Figure 2:**
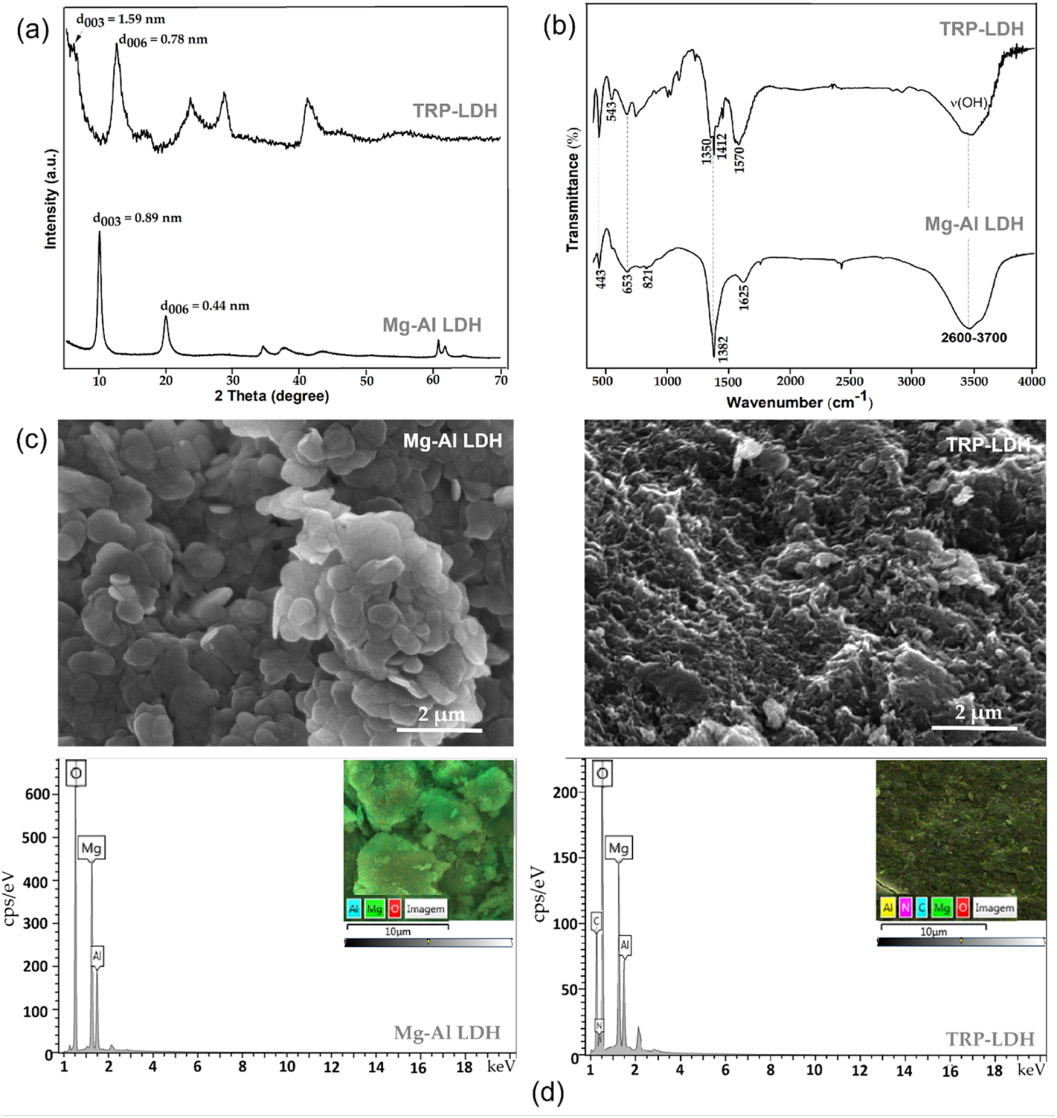
Microstructural and compositional analysis of the synthesized materials. (a) X-ray diffraction (XRD) patterns of pure Mg–Al–NO_3_ layered double hydroxide (LDH) and the LDH-TRP hybrid. (b) Fourier-transform infrared (FTIR) spectra of pure Mg–Al–NO_3_ LDH and the LDH-TRP hybrid. (c) The scanning electron microscopy (SEM) image of the LDH-TRP hybrid. (d) SEM-energy-dispersive X-ray spectroscopy (EDS) elemental mapping of the LDH-TRP hybrid.

At this point, focusing on the Mg–Al LDH intercalated with tryptophan (TRP-LDH) sample, we can observe changes of the reflections at 2θ = 5.15° (*d*_003_ = 1.59 nm) and 11.64° (*d*_006_ = 0.78 nm), respectively,^38^ which confirms the successful intercalation of the organic compound (TRP) into the LDH structure. As the (*d*_003_) spacing value was 1.59 nm for the TRP-LDH sample, and assuming a thickness of 0.48 nm for the Mg–Al layered double hydroxide (LDH) (Mg–Al–NO_3_^–^), the interlayer space is calculated as 1.11 nm, within the range previously reported for amino acids intercalated into LDHs.^20,39^

Spectroscopic analysis provided further insights into the structural features of the LDH materials (Figure 2b). Infrared spectra collected in the mid-IR region (400–4000 cm^–1^) reveal vibrational bands associated with both the layered structure and interlayer anions.^40^ The spectrum of Mg–Al–NO_3_^–^ LDH exhibited a strong absorption band at 1382 cm^–1^, assigned to the asymmetric stretching vibration (ν_3_) of nitrate ions with D_3_h symmetry.^41^

Additionally, a broad band between 2600 and 3700 cm^–1^ is observed, indicating O–H stretching vibrations of water molecules in the interlamellar spaces or adsorbed on the LDH. This band is also associated with the vibrational modes of hydroxyl groups present in the lamellar layers.^42^ In the spectrum of pure LDH, a band around 1625 cm^–1^, corresponding to the (δHOH) mode, is attributed to water molecules between the layers, as well as to the vibrational modes of hydroxyl groups in the lamellar layers M–OH (where M represents Mg and Al).^43,44^ The bands associated with the O–M–O and M–O group bonds (where M is Mg or Al), characteristic of LDH and located in the layers, are observed in the range of 443–821 cm^–1^.^12^

In the case of the TRP-LDH sample, represented by spectrum B in Figure 2b, the absorption bands observed at 443 and 821 cm^–1^ confirm the preservation of the LDH layered structure.^45^ The bands at 1570 and 1412 cm^–1^ are attributed to the asymmetric and symmetric stretching vibrations of the carboxylate (COO^–^) group, respectively.^46,47^ In the range of 3414 cm^–1^, an intense band is associated with v­(N–H) of the indole group, representing the symmetric stretching modes of the amino group. This band overlaps with the broad band between 2600 and 3700 cm^–1^, which indicates O–H stretching vibrations of water molecules in the interlamellar spaces or adsorbed on the LDH surface.^46^

The morphologies observed for the pure Mg–Al LDH and the LDH-TRP hybrid samples are consistent with those previously reported for magnesium–aluminum-based LDH clays, typically exhibiting platelet-like structures, as shown in Figure 2c.^9,12,35^ The SEM images reveal that the incorporation of tryptophan anions significantly alters the morphology of the Mg–Al LDH,^48^ resulting in an irregular structure characterized by particle aggregation. This is likely attributed to the adsorption of tryptophan anions onto the positively charged Mg–Al LDH surface, which impedes the complete growth of the particles.^49^ Thus, the XRD and FTIR spectral results are consistent with the SEM and EDX findings.

### 4.2 Drug-Loading Capacity and Release Kinetics

The absorbance of ten standard solutions containing varying concentrations of pure L-tryptophan (5–50 mg L^–1^) was measured by UV–vis spectroscopy in the 200–400 nm range (Figure S1a). Quantification of tryptophan intercalated in the LDH matrix was performed in triplicate at a wavelength of 280 nm. A calibration curve was generated using linear regression, yielding the equation *y* = 0.0229*x* – 0.0081, with a Pearson correlation coefficient (*R* ^2^) of 0.9982. Based on this calibration, the intercalation efficiency of tryptophan into the inorganic LDH matrix was determined to be 53.00 ± 1.92%, with full experimental details provided in the Supporting Information (Figure S1).

The cumulative release profile of TRP from the LDH-TRP hybrid exhibited approximately 51.72% release within the first 15 min and reached complete release (100%) after 360 min under physiological conditions, pH 7.4 (Figure 3A). The greater release observed in the matrix during the first 2 h, followed by a slow release from the second to the 6 h, can be attributed to the release of TRP from deeper interlayer sites, which the increased diffusion distance to the edges, reduces the release rate. This may be further influenced by a possible increase in the rigidity of the layers, as reported in the literature.^50^ Unlike the phenylalanine–LDH system described by Choy et al., which showed sustained release attributed to strong π–π stacking interactions between aromatic rings, the TRP–LDH hybrid lacks such intermolecular stabilization. The absence of strong π–π interactions, coupled with the horizontal orientation of TRP and weaker electrostatic binding, may account for the rapid initial release observed in this system.^51^ Furthermore, the release at pH 7.4 occurs via ion exchange with the ions in the buffer solution, as already reported in the literature.^52,53^

**Figure 3:**
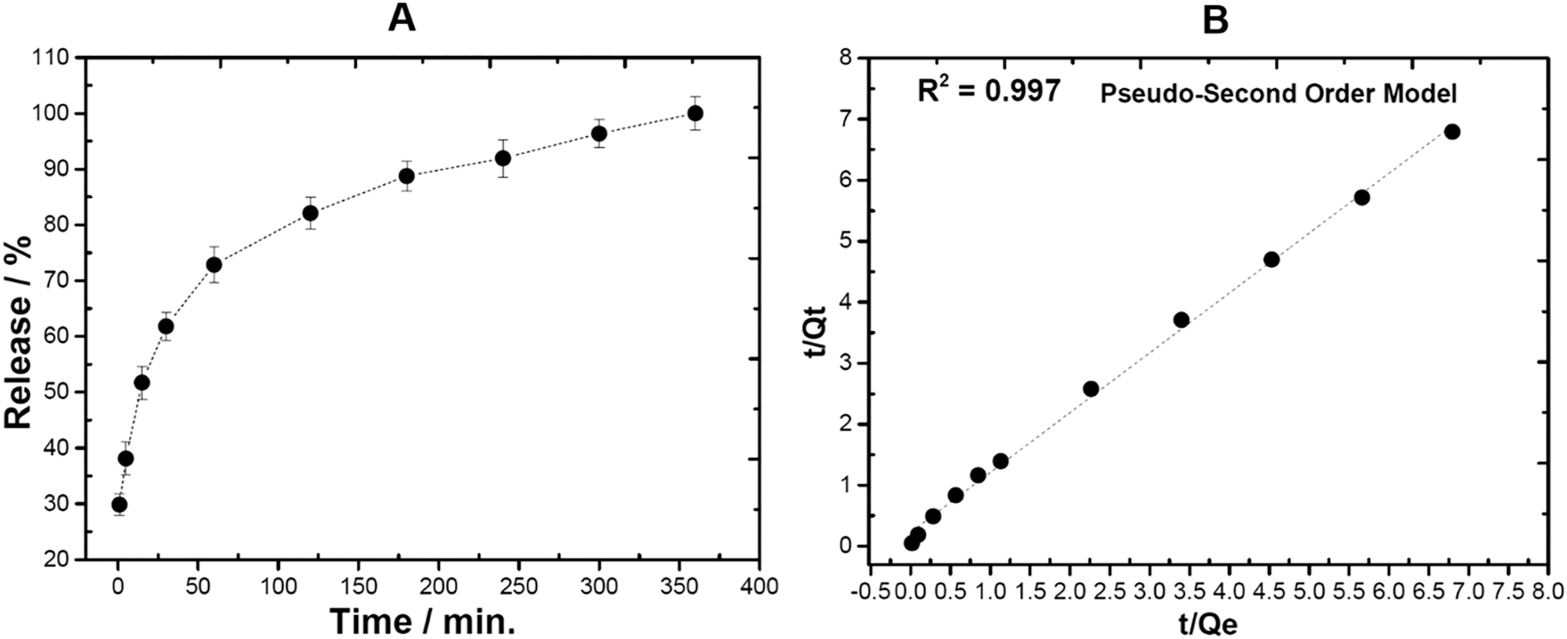
(A) Release profiles for the tryptophan anion from the LDH-TRP sample at pH 7.4. (B) Fitting the data of TRP release from the LDH-TRP sample to pseudo-second order model kinetics in phosphate buffer at pH 7.40.

To corroborate this study, of the seven kinetic models analyzed, first-order, Hixson–Crowell, zero-order, Higuchi, and pseudo-first-order models are not suitable to explain the whole release of hybrid materials (see Table 1). However, the pseudo-first-order model (Figure 3B) was the most satisfactory in our analysis, followed by the Korsmeyer–Peppas model (Table 1). These two models show that a correlation coefficient (R^2^) closer to 1 suggests that the release of TRP is governed by Fickian diffusion due to the concentration gradient between the solid surface and the solution, a release mechanism already observed in Layered Double Hydroxides (LDH). Therefore, this study suggests that the LDH-TRP sample could be a potential drug delivery system.^54^

**Table 1:** Correlation Coefficient (*R* ^2^) Obtained by Fitting Amino Acid Tryptophan (TRP) Release Data from the LDH-TRP Sample into Phosphate-Buffered Saline at pH 7.4

kinetic model	LDH-TRP sample
zero order	*R* ^2^ = 0.746
first order	*R* ^2^ = 0.638
pseudo-first order	*R* ^2^ = 0.937
pseudo-second order	*R* ^2^ = 0.997
Korsmeyer–Peppas^21^	*R* ^2^ = 0.992
Higuchi^22^	*R* ^2^ = 0.911
Hixson–Crowell^53^	*R* ^2^ = 0.676

### 4.3 Behavioral Assays

To test the behavioral effects of the TRP-LDH hybrid, motivational and emotional assays were performed. In the open field test (Figure 4), the pure TRP or the TRP-LDH administration did not modify the motor parameters of center or periphery time or traveled distance. These results suggest that neither 50 mg/kg of pure tryptophan nor tryptophan intercalated in the TRP-LDH system exerted anxiolytic effects in the open field paradigm.

**Figure 4:**
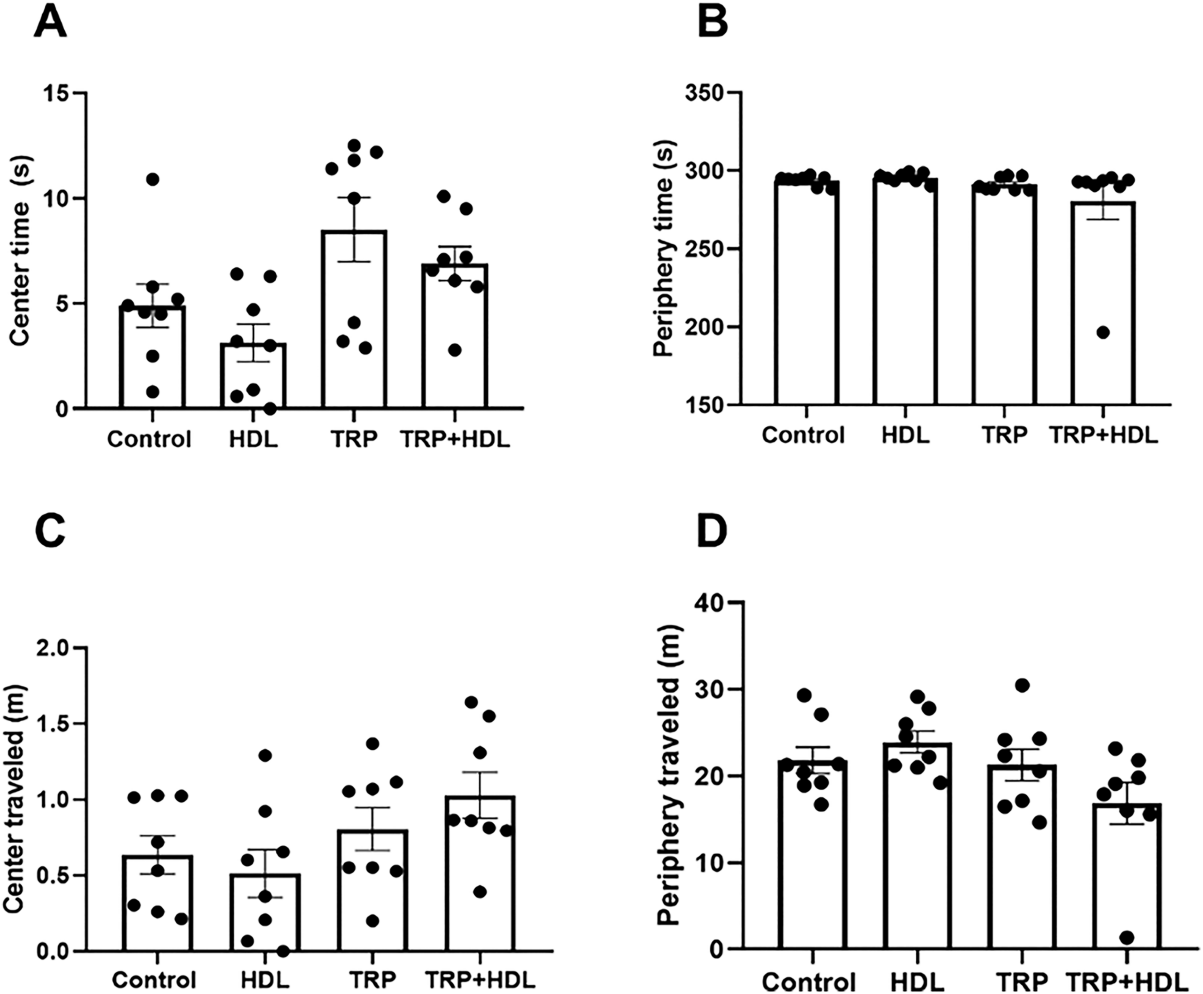
Effect of oral one single dose of intercalated tryptophan solubilized in corn oil (TRP+LDH; 94 mg/kg, equivalent to 50 mg/kg of the pure tryptophan), pure tryptophan (TRP; 50 mg/kg), LDH (1 mL/kg), or corn oil (1 mL/kg; control group) on (A) center time (s); (B) periphery time (s); (C) center distance traveled (m); (D) periphery distance traveled (m) on the open field test. Values are expressed as mean ± SEM (8 animals/group) (Kruskal–Wallis test followed by Dunn’s multiple comparisons test for center and periphery time, and periphery traveled. One-way ANOVA followed by Sidak’s post-hoc test for center distance traveled.

To confirm the absence of the effects on anxiety-type behavior, we employed the elevated plus maze model (Figure 5). In this test, animals that received pure tryptophan or intercalated tryptophan did not show any alterations in performance related to the %EBA, %TBA, or anxiety index parameters (Figure 5A–C). These findings are consistent with the results observed in the open field test, in which neither pure nor intercalated tryptophan displayed anxiolytic effects in the behavioral paradigms.

**Figure 5:**
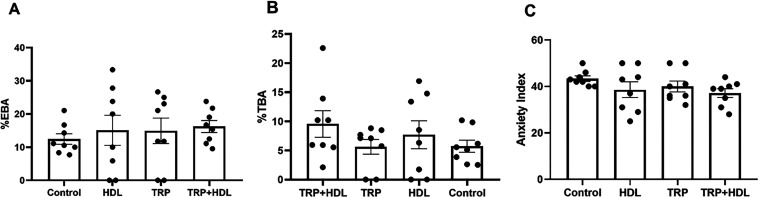
Effect of a single oral dose of intercalated tryptophan solubilized in corn oil (TRP-LDH; 94 mg/kg, equivalent to 50 mg/kg of the pure tryptophan), pure tryptophan (TRP; 50 mg/kg), LDH (1 mL/kg), or corn oil (1 mL/kg; control group) on (A) percentage of open arms entries (%OAE); (B) percentage of open arms time (%OAT); (C) the anxiety index on the elevated plus maze test. Values are expressed as mean ± SEM (8 animals/group) (Kruskal–Wallis test followed by Dunn’s multiple comparisons test for %TBA. One-way ANOVA followed by Sidak’s post-hoc test for %EBA and anxiety index).

The relationship between the amino acid TRP and anxiety is not well understood.^3,22^ A systematic review showed that tryptophan supplementation elicited anxiolytic effects and augmented positive mood behavior in healthy subjects.^3^ Although TRP is a substrate for serotonin synthesis, acute tryptophan depletion in humans is not capable of eliciting anxiety symptoms, whereas preclinical studies have shown that reduced brain tryptophan levels result in anxiogenic effects.^55,56^ In the present study, preclinical tryptophan supplementation did not present anxiolytic effects in adult animals. Such controversial results may be linked to the one single dose, concentration of TRP (50 mg), and the age of animals.

In the splash test, our results demonstrated that pure or intercalated tryptophan did not modify the latency to start grooming or self-cleaning (*p* > 0.05; Figure 6A,B).

**Figure 6:**
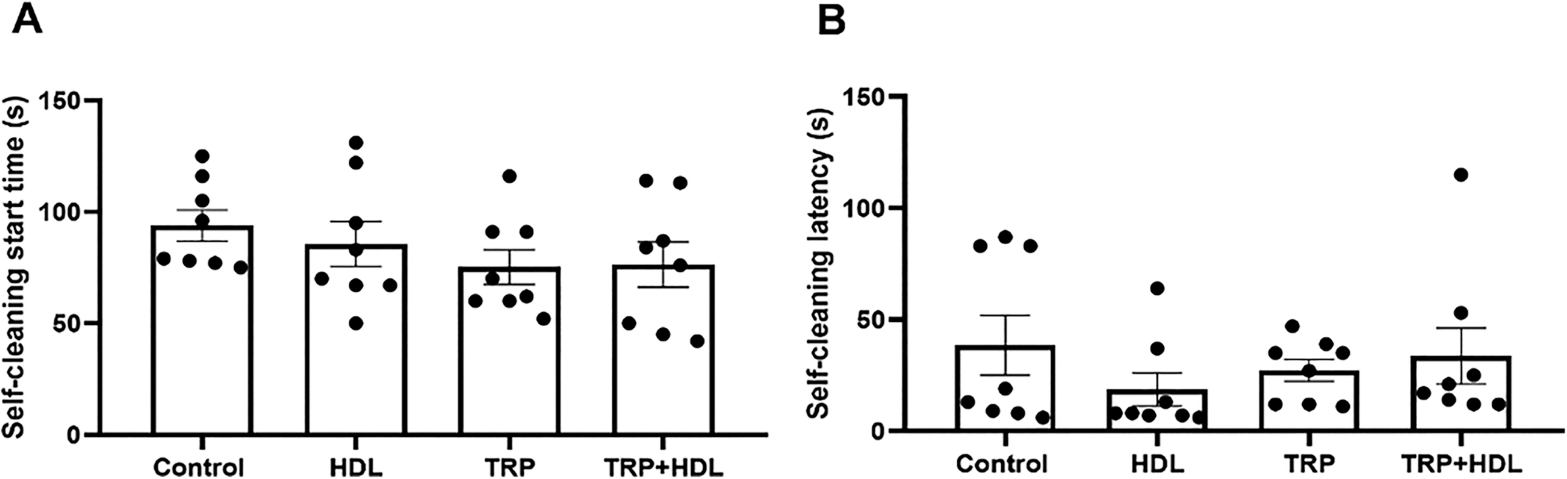
Effect of oral one single dose of intercalated tryptophan solubilized in corn oil (TRP-LDH; 94 mg/kg, equivalent to 50 mg/kg of the pure tryptophan), pure tryptophan (TRP; 50 mg/kg), LDH (1 mL/kg), or corn oil (1 mL/kg; control group) on (A) self-cleaning start latency (s); and (B) self-cleaning latency (s) on the splash test. Values are expressed as mean ± SEM (8 animals/group) (Kruskal–Wallis test followed by Dunn’s multiple comparisons test for self-cleaning latency, and one-way ANOVA followed by Sidak’s post-hoc test for self-cleaning start latency).

The splash paradigm is related to the anhedonia profile. Anhedonia is a core symptom of depression and other psychiatric disorders, in which tryptophan and its metabolites play a crucial role.^57,58^ Beyond serotonin, ∼90% of the tryptophan is converted into kynurenine, which presents several effects on the brain, including anhedonia.^55,57^ Studies have linked fine adjustment in mood disorders related to anhedonia.^57^ Dopamine and γ-aminobutyric acid (GABA) systems are the main neurotransmitters in the reward circuitry involved in anhedonia; however, serotonin contributes to the hedonic state.^58^ Actually, the serotonin from the midbrain raphe nuclei acts on reward-related brain structures (i.e., ventral tegmental area), improving anhedonic behavior through serotonergic neuromodulation along with glutamate and dopamine regulation.^58^ Our absence of the effects on the antianhedonia parameters may be related to multiple neurotransmitters involved in the neurobiology of anhedonia, not only serotonin. This fact may limit the antianhedonia effect of TRP.

The forced swimming test that evaluates antidepressant effects of compounds,^30,32^ was employed to verify antidepressant effects of TRP (Figure 7). Interestingly, animals that received 50 mg of pure tryptophan reduced the immobility time (*p* < 0.05; Figure 7F), but not the frequency and time of struggling and swimming, as well as the immobility frequency (Figure 7A–E). However, the group that received the intercalated tryptophan additionally improved parameters related to antidepressant features, including struggle frequency and time, and immobile frequency. Besides, in the struggling behavior parameters (Figure 7A,B), the animals that received LDH-TRP administration exhibited more efficient performance than the pure tryptophan subjects (*p* < 0.05). These findings highlight the functional superiority of the LDH-TRP system over free TRP in eliciting antidepressant-like responses, consistent with previous evidence from Ferreira Meneses et al.,^9^ where LDH-intercalated indomethacin exhibited enhanced efficacy over the free drug in attenuating neuroinflammatory symptoms. All of these pieces together suggest that the intercalated tryptophan probably improved the delivery of this essential amino acid for the brain, increasing the levels of the brain’s serotonin and motivational features related to depression symptoms.

**Figure 7:**
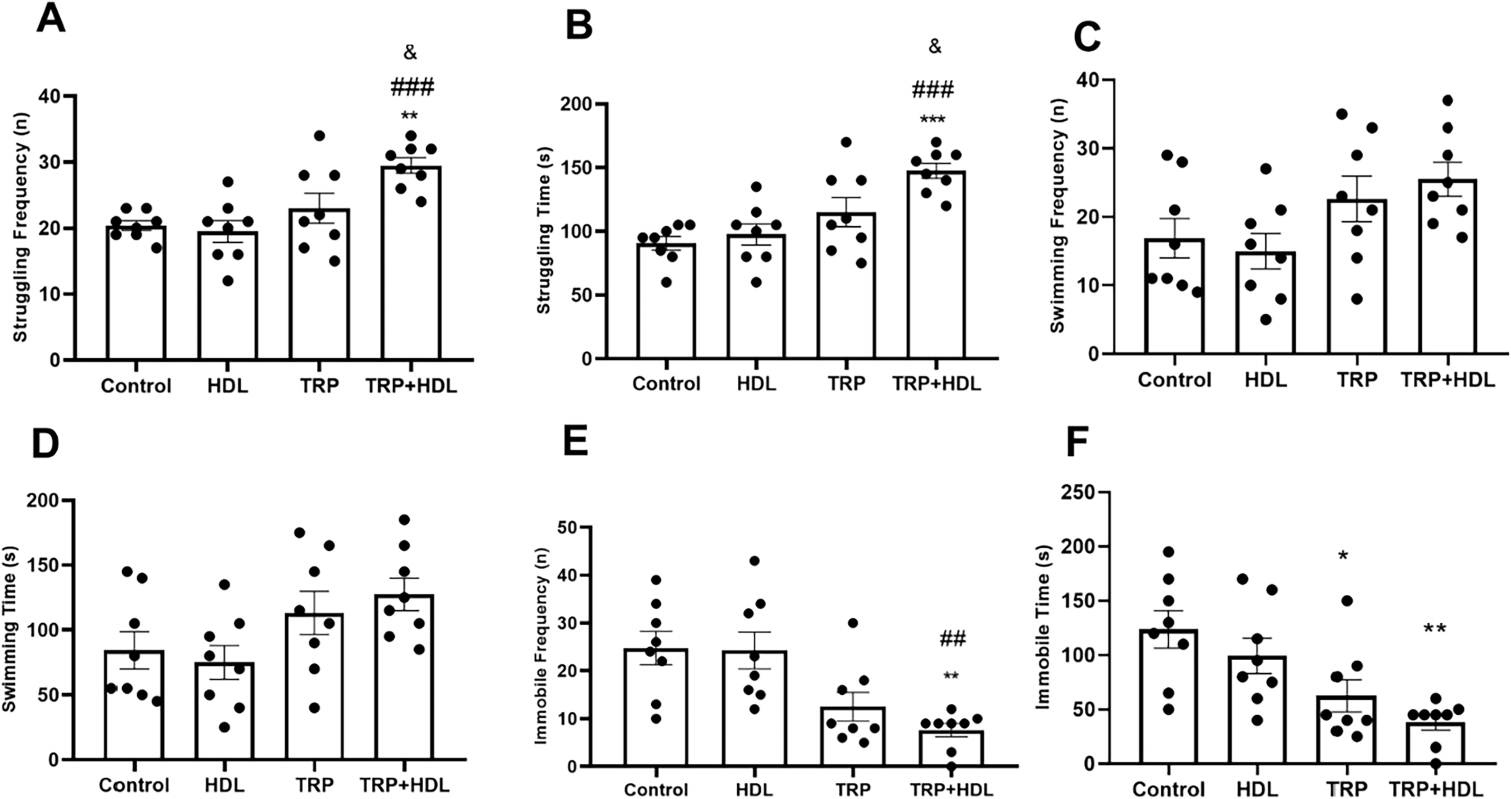
Effect of an oral one single dose of intercalated tryptophan solubilized in corn oil (TRP-LDH; 94 mg/kg, equivalent to 50 mg/kg of the pure tryptophan), pure tryptophan (TRP; 50 mg/kg), LDH (1 mL/kg), or corn oil (1 mL/kg; control group) on (A) struggling frequency (*n*); (B) struggling time (s); (C) swimming frequency (*n*); (D) swimming time (s); (E) immobile frequency (*n*); and (F) immobile time (s) on the elevated plus maze test. Values are expressed as mean ± SEM (8 animals/group) (Kruskal–Wallis test followed by Dunn’s multiple comparisons test for struggling and swimming frequency and time, and one-way ANOVA followed by Sidak’s post-hoc test for immobile frequency and time).

The relationship between tryptophan and depression has been well-documented.^3^ Pharmacokinetic studies reveal that tryptophan reaches saturable states at doses above 50 mg/kg.^59^ To assess the central nervous system, tryptophan needs to be an unbound molecule and the amino-acid transporter system, which is also responsible for the transport of several other essential amino acids. Such conditions limit the tryptophan levels in the brain, with consequent reduction of serotonin synthesis.^55^ We hypothesized that intercalated tryptophan facilitates blood–brain barrier passage, resulting in higher central tryptophan levels compared to pure tryptophan, thereby enhancing its antidepressant effects.

These behavioral results suggest the efficiency of the Mg–Al LDH that emerges as a useful tool to improve the delivery of substrates for tissue targets that present biological barriers, such as the central nervous system. In summary, TRP-LDH exhibited a well-characterized profile with effectiveness in the behavioral assays for depression-like phenotypes. However, further complementary studies are needed to clarify the pharmacokinetic parameters of the carrier system and the mechanisms related to the delivery of the substrate across biological barriers.

## 5 Conclusion

In this study, we successfully synthesized and characterized an Mg–Al LDH intercalated with tryptophan (TRP-LDH), confirming the preservation of the lamellar structure through XRD, FTIR, and SEM-EDS analyses. The XRD patterns revealed a decrease in basal spacing from 0.89 nm in the pure LDH to 0.78 nm in the TRP-LDH, indicating a horizontal arrangement of tryptophan molecules, while a low-angle reflection at 1.59 nm suggested a partial vertical orientation. FTIR spectra confirmed the presence of characteristic vibrational bands of the LDH structure and the successful intercalation of tryptophan (3412, 1639, 1421, 1016–735 cm^–1^), with distinct bands for carboxylate and indole groups observed. Morphological analysis by SEM showed significant changes after intercalation, with particle aggregation indicative of surface interactions between tryptophan and the LDH. The intercalation efficiency of tryptophan was determined to be 53.00 ± 1.92%, and its release from the TRP-LDH matrix reached approximately 51.72% in the first 15 min, with complete release after 360 min under physiological pH (7.4). The drug release kinetics best fit the pseudo-second-order model (*R* ^2^ = 0.997), suggesting that the release process may involve chemisorption and is influenced by the reduced crystallinity of the hybrid material. In behavioral assays, administration of TRP-LDH and pure TRP did not significantly affect locomotor or anxiety-like behaviors in the open field and elevated plus maze tests, nor did they alter responses in the splash test, indicating no observable anxiolytic or antianhedonic effects at the tested dose. However, in the forced swimming test, animals treated with pure TRP (50 mg/kg) exhibited a significant reduction in immobility time, while TRP-LDH administration resulted in more pronounced antidepressant-like effects, including increased struggling frequency and time and reduced immobility frequency. These findings suggest improved bioavailability and brain delivery of tryptophan when intercalated in the LDH matrix. Altogether, the results demonstrate that TRP-LDH maintains structural integrity, achieves moderate intercalation efficiency, provides controlled release, and enhances antidepressant-like behavioral outcomes compared to free tryptophan, supporting its potential as a promising platform for delivering amino acids to target neuropsychiatric conditions. These findings prove that intercalating l-tryptophan into layered double hydroxides can overcome key pharmacokinetic limitations and improve the CNS bioactivity. Given its biocompatibility, controlled release behavior, and behavioral efficacy, the LDH-TRP system holds significant translational potential for development as a nutraceutical intervention or as part of an advanced CNS-targeted drug delivery platform. The limitation of the present study consists of the absence of liver and kidney toxicological assessments, which deserve further attention.

## Supplementary Material



## Notes

Equation used for the calculation of TRP loading; calibration curve of pure TRP; UV–Vis spectra in the 200–400 nm range; and determination of the intercalation efficiency of TRP into the LDH matrix. (PDF)
