# Supplementary material for: Mg–Al LDH/Tryptophan Nanoparticle System Improves Depressive-like Behavior in Rats
Source: ACS Omega. 2025 Sep 15;10(37):43219–29. doi: 10.1021/acsomega.5c06491 (PMC12461313; doi:10.1021/acsomega.5c06491)
Supplement: Supplementary file 1 [file ao5c06491_si_001.pdf]

Supplementary Information Section

Mg-Al LDH/Tryptophan Nanoparticle System Improves Depressive-Like Behavior in Rats

Juliana P. S. Nascimento[a,b]; Taiana C. V. S. Carvalheiro-Simas[c]; Arnold Ferreira Janssen[a]; Maria Luane de S. da Silva[c]; Juliana C. Valente[a]; Mario L. Barreto de Jesus[a]; Flávia G. Silva[d], Enéas A. Fontes-Júnior[c]; Waldeci Paraguassu[e]; Cláudio M. R. Remédios[e]; Kelson do Carmo Freitas Faial[f]; Paulo R. M. Sousa[a,g]; José Rogério A. Silva[g,h,i]; Jerônimo Lameira[g,h]; Cristiane S. F. Maia[c]*; Carla Carolina F. Meneses[a,g] and Cláudio N. Alves[a,b,g]

[a]Laboratory of Natural Resources and Sustainability of the Amazon, Institute of Exact and Natural Sciences, Federal University of Pará, Belém 66075-110, Pará, Brazil

[b]Graduate Program in Medicinal Chemistry and Molecular Modeling, Institute of Health Sciences, Federal University of Pará, Belém 66075-110, Pará, Brazil

[c]Laboratory of Pharmacology of Inflammation and Behavior, Institute of Health Sciences, Federal University of Pará, Belém, Pará, Brazil

[d]Federal University of Western Pará, Institute of Public Health, Santarém 68035-110, Pará, Brazil

[e]Laboratory of Nanoscience and Nanotechnology of the Amazon, Faculty of Physics, Federal University of Pará, Belém 66075-110, Pará, Brazil

[f]Evandro Chagas Institute, Ministry of Health, Ananindeua 67030-000, Pará, Brazil

[g]Graduate Program in Sciences and Environment, Institute of Exact and Natural Sciences, Federal University of Pará, Belém 66075-110, Pará, Brazil

[h]Laboratory of Computer Modeling of Molecular Biosystems (CompMBio), Federal University of Pará, Belém 66075-110, Pará, Brazil

[i]Catalysis and Peptide Research Unit, University of KwaZulu-Natal, Durban 4000, South Africa

Estimation of the TRP loading

For the determination of the drug loading in the LDH-TRP sample was performed as described in our previous works [22,23]. In summary, an average of 1mg of LDH-TRP were dissolved in 0.5 ml of pure ethanol and 0.5 ml of 0.1 M HCl solution, diluted with phosphate buffer (pH 7.40) solution in a 10 ml volumetric flask, and then the solution was analyzed using a UV–Vis spectrophotometer (Shimadzu UV-2600) at λ = 280 nm. The percentage of TRP was calculated following equation (1):

$$\% \, drug \, loading = \frac{amount \, of \, TRP \, (mg)}{amount \, of \, LDH - TRP \, hybrid \, (mg)} \, x \, 100 \qquad (1)$$

The calibration curve, from the pure TRP, showed a correlation coefficient of 0.9982, governed by the equation (y = 0.0229x – 0.0081).

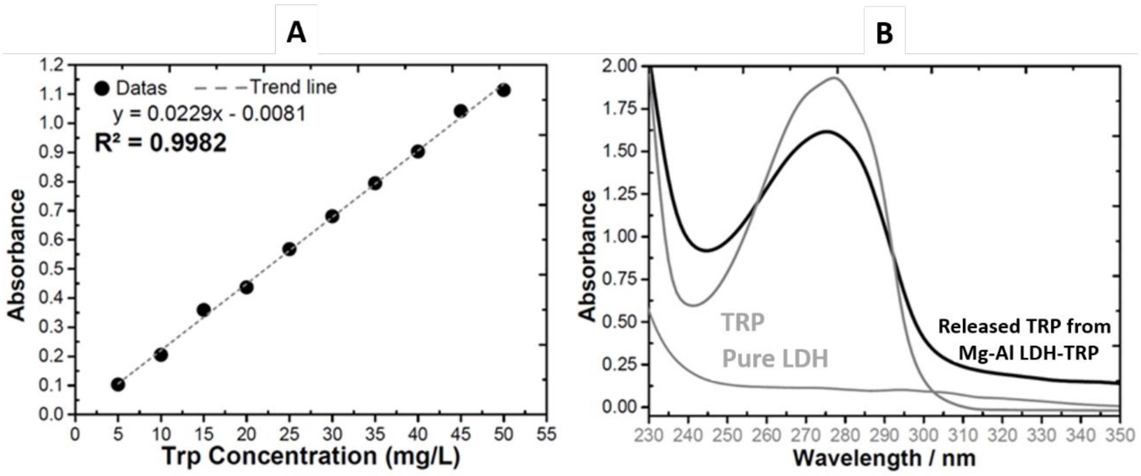

Figure S1. (a) The calibration curve obtained from a series of standard solutions of pure L-tryptophan (5-50 mg L^{-1}) measured by UV-vis spectroscopy in the 200-400 nm range. (b) The intercalation efficiency of tryptophan into the inorganic LDH matrix was determined to be 53.00% ± 1.92%.
